# Observational study on patterns of neuromuscular blockade reversal

**DOI:** 10.1186/s12871-016-0266-2

**Published:** 2016-10-22

**Authors:** Timur Dubovoy, Michelle Housey, Scott Devine, Sachin Kheterpal

**Affiliations:** 1 Department of Anaesthesiology, University of Michigan Medical School, CVC 4172, 1500 East Medical Centre Drive, Ann Arbour, MI 48109 USA; 2 Merck, Sharp, and Dohme, Inc, P.O. Box 100, Whitehouse Station, NJ 08889-0100 USA

**Keywords:** Neuromuscular blockers, Neostigmine, Neuromuscular blockade reversal

## Abstract

**Background:**

Using electronic health record data, we hypothesized that larger reversal doses are used for patients with deeper levels of neuromuscular blockade (NMB) as evidenced by the last recorded TOF measurement. We also examined if dosing regimens reflect current practice guidelines of using ideal body weight (IBW) for NMB agents and total body weight (TBW) for neostigmine.

**Methods:**

This is a retrospective observational study of adult, ASA 1–4 patients who underwent general anaesthesia and received non-depolarizing NMB agents between 01/01/2004 and 12/31/2013. For the primary outcome, percentages of cases receiving neostigmine and median doses administered for each subjective train-of-four (TOF) category were calculated. Secondary analyses evaluated associations between NMB dosing and neostigmine administration based on Body Mass Index (BMI) categories.

**Results:**

A total of 135,633 cases met inclusion criteria for the study. There was no clinically significant difference in median neostigmine dosing based on last TOF count prior to reversal administration: 37.5 mcg/kg for TOF of 4/4 vs. 37.9 mcg/kg for TOF of 0/4 for the total neostigmine dose. Significantly higher number of patients with lower TOF counts received additional neostigmine administration: 5.7 % for 0/4 vs. 1.5 % for 4/4 TOF counts. The median times to extubation following neostigmine administration were clinically similar across TOF count categories. The median doses for neostigmine based on TBW decreased with higher BMI categories and were significantly different between the lowest and highest categories: 42.8 mcg/kg vs 30.8 mcg/kg for total doses (*p* < .0001) respectively. The percentages of cases requiring reversal in addition to the initial dose increased with increasing BMI categories and were 2.1 % for BMI < 18 vs. 3.3 % for BMI ≥ 40. The total median dose of NMB agents in ED95 equivalents per IBW increased from 2.9 in the Underweight category to 4.2 in the Class III Obese category. The majority of patients in the pancuronium subgroup received very low ED95 equivalent dose of 0.1 and did not require reversal. Patients receiving cisatracurium were given significantly higher median ED95 equivalent dose of 5.6 vs 2.8–3.9 compared to other intermediate acting NMB agents, while receiving clinically similar doses of neostigmine.

**Conclusions:**

Neither neostigmine dosing nor times to extubation were affected by the depth of the neuromuscular blockade prior to reversal. The need for additional reversal, or rescue, correlated strongly with the depth of NMB. There was significant variability in neostigmine dosing across the BMI categories. Underweight patients received relatively lower NMB doses while simultaneously receiving relatively higher reversal doses, and the opposite was true for patients with BMI >40.

## Background

Non-depolarizing neuromuscular blockade (NMB) is commonly used to facilitate tracheal intubation and surgical conditions in patients undergoing general anaesthesia. It remains a mainstay of a balanced anaesthetic technique despite advances in short acting volatile and intravenous anaesthetics. Our understanding of the prevalence and impact of residual postoperative NMB has been greatly expanded in recent years [1, 2]. Even mild levels of residual NMB decrease objective measures of pulmonary function, increase the risk of aspiration and airway obstruction, and worsen patient recovery experience [3–6]. Residual neuromuscular blockade continues to be a widespread problem, affecting greater than 50 % of patients receiving NMB in normal practice, even despite qualitative neuromuscular monitoring and the use of neostigmine [7].

Although overwhelming majority of anaesthesiologists surveyed in Europe and the United States believe that either subjective or quantitative train-of-four (TOF) monitoring may improve NMB management [8], neither modality has been accepted as standard of care for patients receiving neuromuscular blocking agents. Among reasons cited for this lack of adoption of monitoring standards is significant practice variation in NMB management that exists between countries, hospitals, and individual practitioners [8, 9]. For example, according to Naguib et al. [8], “surveys in Denmark, Germany, the United Kingdom, and Mexico have suggested that only 43, 28, 10, and 2 % of clinicians respectively, routinely use neuromuscular monitors of any kind.” In addition to practice variations in NMB monitoring, there are significant differences in the use of reversal agents at the end of a surgical procedure. In their response to a survey, 82 % of European and 65 % of American practitioners have reported that they did not routinely administer a reversal agent following use of non-depolarizing NMB drugs [8]. Current experts’ opinions suggest that NMB monitoring should guide administration of NMB agents [1, 4] as well as NMB reversal agents [10, 11]. However, a wide gap continues to exist between experts’ recommendations and current clinical practice of monitoring and NMB antagonism [9, 12]. Presently available data on patterns of clinical practice are mainly limited to small (<1000 patients) observational studies [13] and survey results [8]. There are no recent data describing the routine practice patterns of reversal and its relationship to TOF monitoring. A better understanding of practice patterns is needed in order to guide future recommendations and target efforts to improve clinical practice and patient safety.

We used a large, granular intraoperative health record dataset to identify current practices in neostigmine reversal. The primary objective of this study was to assess whether dosing of neostigmine is related to the use of subjective train-of-four (TOF) monitoring. We hypothesized that reversal dosing is based upon the last recorded subjective TOF assessment, with larger doses used for patients with deeper levels of NMB as evidenced by lower TOF counts. We also examined whether dosing regimens reflect current practice guidelines of using ideal body weight (IBW) for NMB agents [6, 14–16] and total body weight (TBW) for neostigmine [14, 17, 18].

## Methods

### Study design and setting

This retrospective observational study of adult, ASA 1–4 patients who underwent general anaesthesia and received non-depolarizing NMB agents at the University of Michigan between 01/01/2004 and 12/31/2013. This study received approval from the University of Michigan Institutional Review Board (HUM00091819). The informed consent was waived since all identifiable patient elements were removed prior to data analysis. We excluded patients who were intubated prior to OR arrival, patients transported to ICU following surgical procedure, cardiac surgery, lung or liver transplantation, cases where neostigmine was administered to facilitate intraoperative neurologic monitoring with subsequent re-dosing of NMB agents, and patients with myasthenia gravis or those receiving pyridostigmine therapy. Monitoring of the neuromuscular blockade and TOF counts were measured using the MiniStim® MS-IV (Life-Tech, Stafford, TX) peripheral nerve stimulator. All data for this study was gathered from the local University of Michigan Health System (UMHS) anaesthesia information management system (AIMS) and electronic health record (EHR) (Centricity®, General Electric Healthcare, Waukesha, WI). Basic patient anthropometrics, including patient age, gender, body mass index (BMI), ASA classification, emergent classification, and procedural information including case duration and surgical service were extracted from the EHR. In addition, we collected dose and time of administration information for neostigmine and all non-depolarizing NMB drugs: vecuronium, rocuronium, atracurium, cisatracurium, and pancuronium. We converted the doses of neuromuscular blockers to effective doses required to reduce the maximum twitch height by 95 % in 50 % of the population (ED95 equivalents), corrected for ideal body weight [6, 19, 20]. The following conversions were used: vecuronium 0.05 mg/kg, rocuronium 0.3 mg/kg, atracurium 0.26 mg/kg, cisatracurium 0.05 mg/kg and pancuronium 0.07 mg/kg. Weight correction for neostigmine was performed using total body weight (TBW) [14, 17, 18]. Extubation times for all included cases were extracted as well.

### Statistical analysis

All statistical analyses were performed using SPSS® version 21 (Armonk, NY) and SAS® software, version 9.3 (Cary, NC). Missing data for each covariate were evaluated - only case duration was missing more than 10 % (21.1 %). Patients with missing or invalid height or weight values were categorized as missing for the BMI variable (3.5 %). Undocumented TOF data were analysed as a separate category. To assess current usage of neostigmine, patient and case characteristics were summarized with frequency counts and percentages. Age was normally distributed and reported as means and standard deviations, while surgical case duration (minutes) was not normally distributed and reported as medians and interquartile ranges. Overall trend in neostigmine administration and subjective train-of-four (TOF) documentation was examined quarterly during the study period. For the primary objective, percentages of cases receiving neostigmine (initial dose, second dose, third dose and total dose) and median doses administered for each subjective train-of-four (TOF) category were calculated, as well as minutes between NMB dose, neostigmine administration and extubation. The relationship between NMB dose in ED95 equivalents and TOF categories were also examined. Correlation coefficients, Pearson chi-square tests and Mann-Whitney tests quantified associations and evaluated statistical significance. A *p*-value of <0.05 was considered to be statistically significant. Secondary analyses evaluated associations between NMB dosing and neostigmine administration based on World Health Organization Body Mass Index (BMI) categories.

## Results

**Fig. 1:**
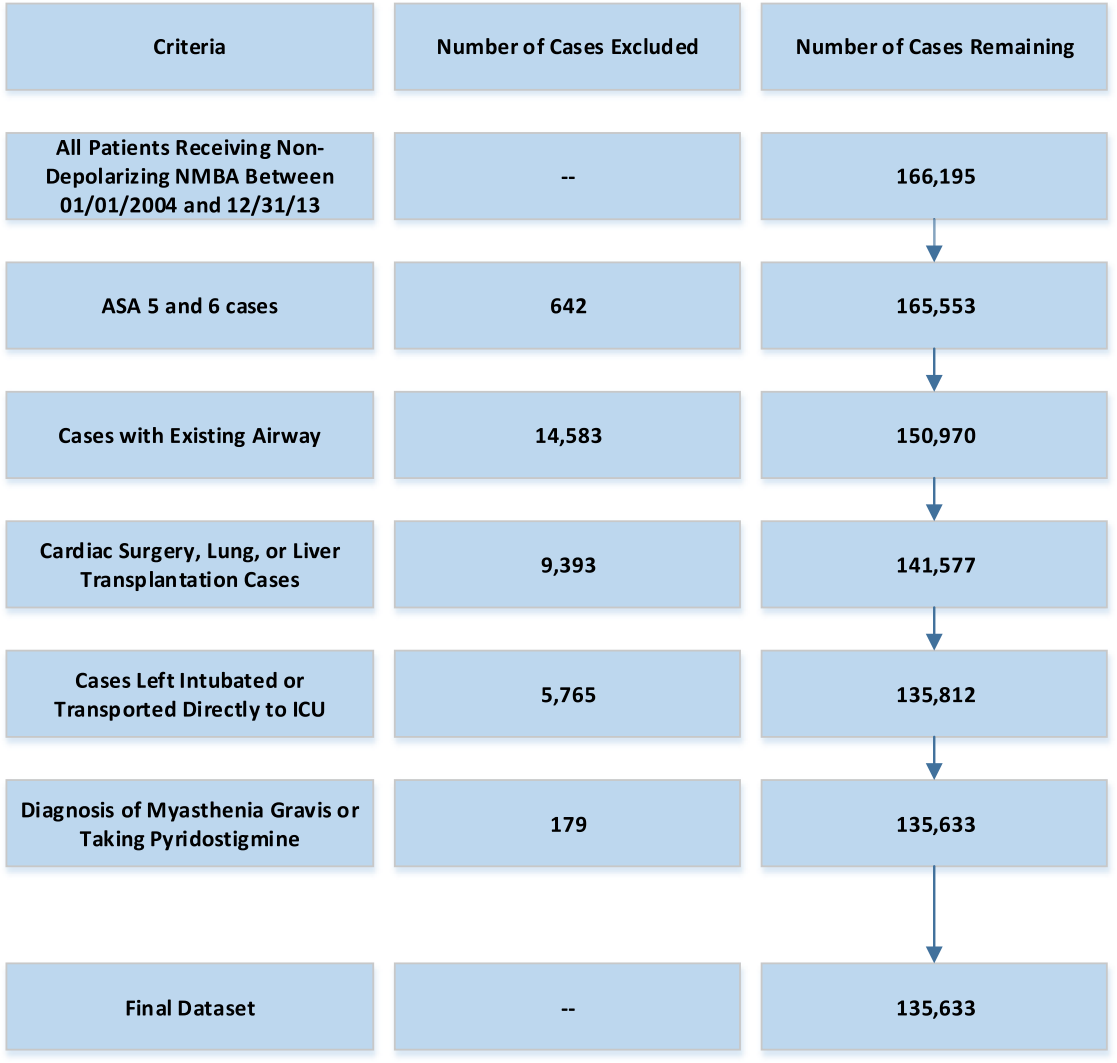
Study Population Breakdown. NMDA = Neuromuscular Blocking Agent

**Fig. 2:**
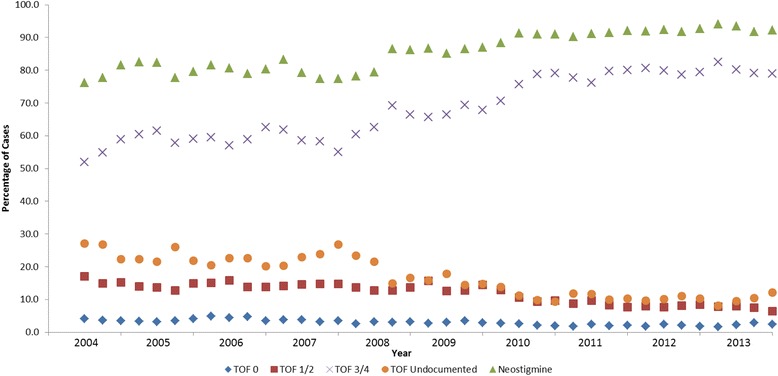
Trend in Neostigmine use and Train of Four (TOF) documentation by quarter, May 2004 - December 2013

Table 1 shows patient and case characteristics for the final 135,633 cases that met inclusion criteria from the total of 166,195 cases studied. The stepwise exclusion process is shown in Fig. 1. Monitoring of the neuromuscular blockade with subjective tactile TOF count has been employed and documented in 83.9 % (113,869), and reversal of NMB with neostigmine occurred in 86.4 % (117,123) of all cases that used non-depolarizing NMB agents. Of the 18,510 cases who did not receive neostigmine, 69.7 % had no documentation of monitoring. Contrary to our original hypothesis, there was no clinically significant difference in median neostigmine dosing based on last TOF count prior to reversal administration: 37.5 mcg/kg [range 3.8–125 mcg/kg] for TOF of 4/4 vs 37.0 mcg/kg [range 5.0–95.7 mcg/kg] for TOF 0/4 for the initial neostigmine dose and 37.5 mcg/kg for TOF of 4/4 vs. 37.9 mcg/kg for TOF of 0/4 for the total neostigmine dose. However, significantly higher number of patients with lower TOF counts received additional neostigmine administration: 5.7 % for 0/4 TOF, 5.2 % for 1/4 TOF, 3.6 % for 2/4 TOF, 2.5 % for 3/4 TOF, and 1.5 % for 4/4 TOF counts (combined 2^nd^ and 3^rd^ neostigmine doses). In addition to the 3.8-fold difference in extra reversal between highest and lowest TOF categories (*p* < .0001), patients with TOF of 4/4 received a significantly lower median neostigmine dose of 14.7 mcg/kg [range 3.4–60.6 mcg/kg] compared to 20.0 mcg/kg [range 5.6–61.3 mcg/kg] for patients in the TOF 0/4 category (*p* < .0001) (based on 2^nd^ neostigmine dose only). These results are summarized in Table 2. There was little correlation between the total dose of NMB agents in ED95 equivalents and TOF categories: 3.5 for TOF of 0/4 and 4/4 and 4.0 for TOF 1–3/4 (r = −0.08795). The median times between administration of the first reversal dose and extubation were not clinically significant between TOF categories: 12.7 min for 4/4 TOF and 12.0 min for 0/4 TOF counts. These findings are summarized in Table 3. The trends in neostigmine use and TOF monitoring are shown in Fig. 2 and demonstrate increase in percentage of cases getting reversal from 76.2 % in 2004 to 92.2 % in 2013, as well as a significant increase from 51.9 to 79.0 % in cases with last TOF count of 3 or 4 out of 4 prior to reversal. The overall use of subjective TOF monitoring has increased from 73.0 to 87.8 % over the ten year study period.

**Table 1:** Patient and Case Characteristics

Patient/Case characteristics	Did not receive neostigmine	Received neostigmine
Total *N* = 135,633	*N* = 18,510 (13.6 %)	*N* = 117,123 (86.4 %)
Age^a^	51 ± 18	52 ± 17
Surgical case duration (minutes)^b^	144 [87,238]	113 [69,177]
Emergent surgery	835 (4.5)	6095 (5.2)
ASA status		
ASA class 1 or 2	10,976 (59.3)	68,910 (58.8)
ASA class 3 or 4	7534 (40.7)	48,213 (41.2)
Gender		
Male	9484 (51.2)	63,165 (53.9)
Female	9026 (48.8)	53,957 (46.1)
World Health Organization BMI categories		
Underweight, BMI <18.5	481 (2.7)	2456 (2.2)
Normal Weight, BMI 18.5–24.9	5466 (30.8)	3,2491 (28.7)
Overweight, BMI 25.0–29.9	5509 (31.0)	35,302 (31.2)
Class I Obesity, BMI 30.0–34.9	3346 (18.8)	22,634 (20.0)
Class II Obesity, BMI 35.0–39.9	1645 (9.3)	11,321 (10.0)
Class III Obesity, BMI ≥40	1326 (7.5)	8973 (7.9)
Last Recorded TOF		
0/4	347 (1.9)	3557 (3.0)
1/4	478 (2.6)	6987 (6.0)
2/4	510 (2.8)	7751 (6.6)
3/4	425 (2.3)	7170 (6.1)
4/4	4187 (22.6)	82,457 (70.4)
Undocumented	12,563 (67.9)	9201 (7.9)
Surgical service		
Dental	734 (4.0)	2904 (2.4)
General	2275 (12.3)	20,532 (17.5)
Gynecology	1000 (5.4)	11,742 (10.0)
Neurology	1937 (10.5)	9395 (8.0)
Ophthalmology	443 (2.4)	1630 (1.4)
Orthopedics	2843 (15.4)	18,273 (15.6)
Other	1296 (7.0)	8763 (7.5)
Otolaryngology	3691 (19.9)	8452 (7.2)
Plastics	1492 (8.1)	7814 (6.7)
Radiology	304 (1.6)	2056 (1.8)
Thoracic	533 (2.9)	5754 (4.9)
Transplant	419 (2.3)	4810 (4.1)
Urology	1093 (5.9)	10,657 (9.1)
Vascular	450 (2.4)	4341 (3.7)
Neuromuscular blockade agent^c^		
Atracurium	1435 (7.8)	4528 (3.9)
Cisatracurium	3613 (19.5)	17,892 (15.3)
Pancuronium	1402 (7.6)	657 (0.6)
Rocuronium	1867 (10.1)	7069 (6.0)
Vecuronium	10,720 (57.9)	88,526 (75.6)
Multiple NMBAs	521 (2.8)	1544 (1.3)
Succinylcholine with NMBA	5891 (31.8)	28,608 (24.4)

*ASA* American Society of Anaesthesiologists, *BMI* body mass index, *TOF* train-of-four, *NMBA* neuromuscular blockade agent

^a^Age is represented as mean ± standard deviation

^b^Surgical case duration is non-parametric and presented as median [25^th^ to 75^th^ percentile]

^c^Cases may have received more than one type of neuromuscular blocking agent

**Table 2:** Neostigmine dose by last documented Train of Four (TOF) prior to reversal and Body Mass Index (BMI) category for cases receiving Neostigmine

	Cases receiving first dose	Initial dose (mcg/kg)	Cases receiving second dose	Time between first and second doses (minutes)	Second dose (mcg/kg)	Cases receiving third dose	Time between second and third doses (minutes)	Third dose (mcg/kg)	Total dose (mcg/kg)
	*N* ^a^	Median [IQR]	*N* (%)^a^	Median [IQR]	Median [IQR]	*N* (%)^a^	Median [IQR]	Median [IQR]	Median [IQR]
Last TOF prior to reversal									
0/4	3520	37.0 [30.6, 42.4]	192 (5.5)	6 [2, 12]	20.0 [14.2, 28.1]	7 (0.2)	5 [3, 8]	22.4 [13.0, 23.9]	37.9 [30.9, 43.7]
1/4	6956	37.1 [30.8, 42.8]	338 (4.9)	6 [2, 11]	17.3 [11.6, 24.8]	18 (0.3)	6 [3, 14]	16.8 [13.8, 27.8]	37.8 [31.3, 43.9]
2/4	7713	37.2 [30.9, 42.2]	267 (3.5)	7 [2, 13]	15.8 [10.8, 25.0]	11 (0.1)	7 [2, 9]	11.1 [8.1, 18.1]	37.6 [31.1, 42.7]
3/4	7133	36.8 [30.7, 41.9]	166 (2.3)	5 [1, 12]	14.5 [10.1, 22.3]	11 (0.2)	7 [3, 10]	14.7 [10.6, 17.9]	37.0 [30.8, 42.3]
4/4	81,958	37.5 [30.6, 41.7]	1136 (1.4)	5 [1, 12]	14.7 [9.9,22.1]	73 (0.1)	3 [1, 6]	11.3 [9.3, 15.2]	37.5 [30.6, 41.7]
Undocumented	9105	34.5 [29.4, 41.1]	217 (2.4)	6 [2, 11]	17.9 [11.1, 26.7]	10 (0.1)	9 [4, 16]	14.1 [11.6, 17.5]	35.0 [29.4, 41.5]
WHO BMI category^b^									
Underweight	2456	42.6 [36.6, 49.2]	50 (2.0)	10 [4, 15]	22.7 [19.3, 31.3]	1 (0.0)	15 [15, 15]	24.1 [24.1, 24.1]	42.8 [36.8, 50.0]
Normal weight	32,491	39.4 [33.3, 44.4]	574 (1.8)	6 [2, 12]	19.2 [14.4, 29.9]	31 (0.1)	6 [2, 9]	17.2 [14.3, 23.9]	39.7 [33.4, 44.8]
Overweight	35,302	37.5 [31.1, 41.7]	609 (1.7)	6 [1, 11]	15.6 [11.9, 25.5]	42 (0.1)	5 [2, 10]	13.2 [10.8, 20.0]	37.6 [31.3, 41.7]
Class I obese	22,634	35.3 [29.7, 40.4]	488 (2.2)	5 [1, 13]	14.4 [10.1, 22.2]	26 (0.1)	3 [1, 7]	10.5 [9.4, 12.8]	35.7 [29.8, 40.7]
Class II obese	11,321	32.7 [28.1, 39.7]	258 (2.3)	4 [1, 11]	11.9 [9.2, 19.6]	14 (0.1)	2 [1, 5]	10.3 [9.0, 13.6]	33.1 [28.4, 40.0]
Class III obese	8973	30.5 [25.0, 37.0]	284 (3.2)	3 [0, 9]	10.4 [7.5, 16.7]	12 (0.1)	4 [1,11]	8.1 [7.0, 14.3]	30.8 [25.3, 37.5]

*TOF* train-of-four, *WHO* World Health Organization, *BMI* body mass index

^a^Only among patients with TBW between 40 and 250 kg and receiving neostigmine

^b^Only among patients with BMI between 10 and 80

**Table 3:** Neuromuscular Blocking Agent dose by last documented Train-of-Four prior to reversal and Body Mass Index category

	Total dose of NMB (ED 95 equivalent)^a^	Last dose of NMB (ED 95 equivalent)^a^	Time between last NMB & first neostigmine dose (minutes)^b^	Time between first neostigmine dose & extubation (minutes)^b^
	Median [IQR]	Median [IQR]	Median [IQR]	Median [IQR]
Last TOF prior to reversal				
0/4	3.5 [2.4, 5.2]	1.3 [0.7, 2.4]	52.0 [36.0, 80.3]	12.0 [8.0, 18.0]
1/4	4.0 [2.8, 5.8]	0.8 [0.5, 1.7]	42.0 [29.0, 59.0]	12.5 [8.0, 19.0]
2/4	4.0 [2.8, 5.9]	0.7 [0.4, 1.4]	43.6 [31.0, 61.0]	12.8 [8.0, 18.9]
3/4	4.0 [2.7, 5.8]	0.7 [0.4, 1.5]	49.0 [35.0, 68.0]	12.4 [8.0, 19.0]
4/4	3.5 [2.5, 5.3]	0.8 [0.5, 1.8]	65.0 [44.0, 95.0]	12.7 [8.0, 19.0]
Undocumented	3.3 [2.2, 5.1]	0.7 [0.4, 1.8]	43.0 [24.0, 76.0]	12.0 [7.3, 18.0]
WHO BMI category^c^				
Underweight	2.9 [2.0, 4.4]	0.8 [0.5, 1.6]	57.4 [39.0, 86.4]	13.8 [9.0, 21.0]
Normal Weight	3.3 [2.2, 4.9]	0.8 [0.4, 1.8]	58.0 [39.0, 86.0]	13.0 [8.0, 19.0]
Overweight	3.6 [2.5, 6.3]	0.7 [0.4, 1.8]	58.9 [39.0, 87.9]	12.1 [8.0, 19.0]
Class I Obese	3.8 [2.7, 5.7]	0.8 [0.5, 1.9]	59.0 [39.0, 87.5]	12.1 [8.0, 19.0]
Class II Obese	4.0 [2.7, 5.9]	0.8 [0.5, 2.0]	58.5 [39.1, 88.1]	12.3 [8.0, 19.0]
Class III Obese	4.2 [2.9, 6.3]	0.8 [0.5, 1.9]	57.0 [38.0, 85.0]	12.9 [8.0, 19.0]

*ED95* effect dose for which 95 % of the population exhibits the effect, *TOF* train-of-four, *WHO* World Health Organization, *BMI* body mass index

^a^Only among patients with IBW between 40 and 250 kg

^b^Only among patients receiving neostigmine

^c^Only among patients with BMI between 10 and 80

**Fig. 3:**
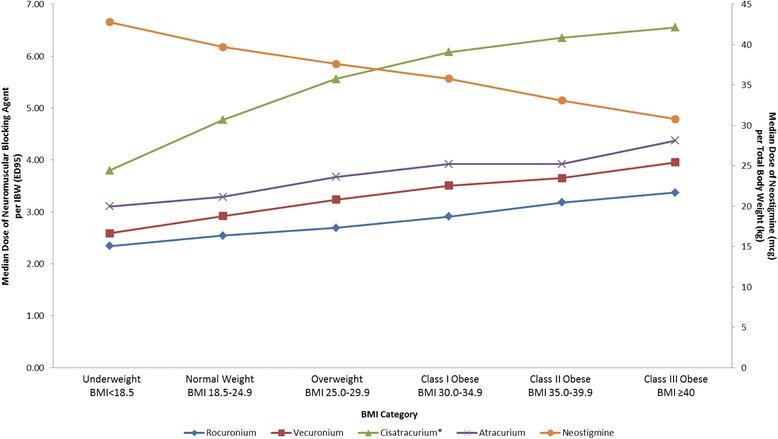
Variation in median dose of intermediate duration neuromuscular blocking agents (NMBs) indexed to Ideal Body Weight (IBW) and Neostigmine dose indexed to Total Body Weight (TBW) across Body Mass Index (BMI) categories [6, 14–17]. * Cisatracurium doses represent the total amount of drug given (infusions and boluses)

The median doses for neostigmine based on TBW decreased with higher BMI categories and were significantly different between the lowest (Underweight, BMI <18.5) and highest (Class III Obese, BMI ≥ 40) categories: 42.6 mcg/kg vs 30.5 mcg/kg for initial doses (*p* < .0001) and 42.8 mcg/kg vs 30.8 mcg/kg for total doses (*p* < .0001) respectively (Table 2). The percentages of cases requiring reversal in addition to the initial dose (2^nd^ and 3^rd^ neostigmine doses combined) increased with increasing BMI categories and were 2.1 % for BMI < 18 vs. 3.3 % for BMI ≥ 40. The total median dose of NMB agents in ED95 equivalents per IBW increased from 2.9 in the Underweight category to 4.2 in the Class III Obese category (Table 3). The variations in the administration of neostigmine and NMB agents across different BMI categories are represented graphically in Fig. 3. The time intervals between initial neostigmine administration and extubation were clinically similar (within one minute) across the BMI categories.

Analysis of subgroups based on individual NMB agents, showed that only 31.9 % of patients receiving pancuronium were reversed with neostigmine, compared to 75.9–89.2 % for other non-depolarizing NMB agents. The median dose of pancuronium in ED95 equivalents was 0.1 in the group that did not receive reversal, likely consistent with practice of precurarization (Table 4). The median reversal dose for patients receiving pancuronium was significantly higher than neostigmine doses for intermediate acting neuromuscular blockers: 47.2 mcg/kg vs 33.7–38.2 mcg/kg. Subgroup analysis also demonstrated that patients receiving cisatracurium had significantly higher median ED95 equivalent dose of 5.6 vs 2.8–3.9 compared to other intermediate acting NMB agents, while receiving clinically similar doses of neostigmine 36 mcg/kg vs 33.7–38.2 mcg/kg (Table 4, no succinylcholine). Similar trend was observed for subgroups receiving cisatracurium and no reversal, both with and without succinylcholine.

**Table 4:** Subgroup analysis of individual neuromuscular blocking agents

		Cases receiving neostigmine				Cases not receiving neostigmine		
	Neuromuscular blocking agent	Cases receiving NMB	NMB total dose (ED 95 Equivalent)^a^	Time between last NMB & extubation (minutes)	Total dose of neostigmine (mcg/kg)^b^	Cases receiving NMB	NMB total dose (ED 95 Equivalent)^a^	Time between last NMB & extubation (minutes)
		*N*	Median [IQR]	Median [IQR]	Median [IQR]	*N*	Median [IQR]	Median [IQR]
No Succinylcholine	Atracurium	2981	3.9 [2.6, 5.9]	67.0 [50.0, 90.0]	33.7 [29.7, 41.7]	906	2.9 [2.1, 4.7]	119.0 [85.0, 174.0]
	Cisatracurium	12,905	5.6 [3.9, 8.3]	73.0 [53.0, 102.0]	36.0 [30.2, 42.4]	2596	4.6 [3.1, 7.1]	136.0 [90.0, 204.0]
	Pancuronium	134	1.3 [0.1, 2.3]	99.5 [62.0, 161.0]	47.2 [35.8, 61.7]	48	0.1 [0.1, 0.1]	210.5 [150.5, 265.0]
	Rocuronium	5602	2.8 [2.2, 4.0]	83.0 [59.0, 117.0]	36.6 [29.8, 41.7]	1510	2.4 [1.8, 3.4]	126.0 [89.0, 174.0]
	Vecuronium	65,708	3.4 [2.4, 4.9]	76.0 [55.0, 108.0]	38.2 [31.5, 42.2]	7343	2.8 [2.0, 4.5]	145.0 [93.0, 233.0]
	Multiple NMBAs	1185	5.9 [4.4, 8.0]	71.0 [52.0, 101.0]	39.0 [32.1, 44.9]	216	6.6 [4.8, 8.6]	92.0 [37.5, 162.5]
Succinylcholine	Atracurium	1407	3.4 [2.2, 5.4]	60.0 [46.0, 84.0]	31.8 [28.2, 39.0]	439	2.5 [1.5, 4.4]	95.0 [65.0, 141.0]
	Cisatracurium	4591	4.8 [2.9, 7.6]	65.0 [49.0, 88.0]	32.7 [28.8, 40.0]	864	4.1 [2.2, 7.5]	106.0 [68.0, 153.0]
	Pancuronium	63	2.1 [1.2, 2.6]	96.0 [74.0, 140.0]	37.0 [31.6, 48.0]	1025	0.1 [0.1, 0.1]	101.5 [60.0, 181.5]
	Rocuronium	674	2.1 [1.3, 3.0]	62.0 [42.0, 87.0]	32.1 [26.1, 39.4]	232	1.0 [0.3, 2.0]	81.0 [53.0, 129.0]
	Vecuronium	21,514	3.0 [1.9, 4.7]	67.0 [49.0, 93.0]	36.7 [30.0, 41.3]	3026	2.6 [1.3, 4.8]	115.0 [67.0, 195.0]
	Multiple NMBAs	359	3.6 [2.2, 5.6]	65.0 [46.0, 94.0]	35.1 [30.0, 41.3]	305	3.0 [1.5, 6.3]	82.0 [41.0, 154.0]

^a^Only among patients with IBW between 40 and 250 kg

^b^Only among patients with TBW between 40 and 250 kg

## Discussion

In this large, single-centre study we demonstrate that contrary to the published guidelines and our hypothesis, there was little correlation between monitoring of the depth of neuromuscular blockade and dosing of neostigmine in clinical practice. The total neostigmine dose tended to be weight-based and fell into a narrow clinical range of 37.0–37.9 mcg/kg across all TOF categories and was significantly lower than the “standard dose” of 50 mcg/kg reported by other sources [12, 19] and recommended for reversing TOF counts between 1 and 3 [10]. Although our single centre database may reflect institutional bias and site-specific clinical care processes associated with a large academic medical centre with an anaesthesiology training program, our median reversal doses were remarkably similar to the mean neostigmine dose of 32 mcg/kg recently reported by Roach and Smith [12].

The median time from reversal administration to extubation also did not change significantly (12.0–12.8 min range) based on the TOF count prior to reversal. Together these findings suggest that when using non-depolarizing NMB agents, the vast majority of providers employed TOF monitoring and administered reversal, however neither neostigmine dosing nor time to extubation were affected by the depth of the neuromuscular blockade prior to reversal. One parameter that correlated strongly with the depth of NMB was the need for additional reversal, or rescue, when initial dose failed to produce desired clinical effect. In addition, 3.0 % of patients received inappropriate reversal when the neostigmine was administered with TOF count of 0/4 [10]. This is markedly lower than reported in recent literature from other single-centre analyses [6]. It was encouraging to see that the overall number of cases receiving clinically-indicated neostigmine has increased throughout the study period, as well as the percentage of cases with TOF counts of 3–4/4. We speculate that this reflects increased awareness of the residual neuromuscular blockade by clinical providers in recent years.

Although median values for neostigmine did not change much based on last TOF counts, there was significant variability in its dosing across the BMI categories. Both categories at the extremes of the BMI spectrum have been shown to be at risk for residual neuromuscular blockade [7] or postoperative pulmonary complications: 6.39 % for BMI < 18 and 4.15 % for BMI > 35 vs. 3.61 % for normal weight patients [6]. A recent study by Sasaki et al. [21] demonstrated that high-dose or unwarranted use of neostigmine may be associated in increased incidence of postoperative respiratory events. Further evidence that reversal with acetylcholine inhibitors may be undesirable in the absence of neuromuscular blockade was provided by Herbstreit et al. [22], supporting the idea that reversal dose must be closely matched to the depth of the existing NMB [10]. Our data suggest that different mechanisms may be responsible for the higher reported incidence of adverse events in underweight and severely overweight patients. As Fig. 3 demonstrates, underweight patients tend to receive relatively lower NMB doses while simultaneously receiving relatively higher reversal doses, potentially placing them at an increased risk from excessive use of acetylcholine inhibitors. The opposite is true for patients with BMI > 40, who may be at higher risk due to overdosing of NMB and relative underdosing of neostigmine. This situation may not be unique to neostigmine, as both recurarization and incomplete reversal with appropriate does of sugammadex have been reported in obese patients receiving high doses of rocuronium [23, 24]. Our findings of potential NMB depth/reversal mismatch across BMI categories raise interesting questions and warrant further investigation through prospective trials.

There are several limitations inherent in this observational analysis of routinely collected intraoperative electronic health record data. First, the analyzed dataset of patient characteristics and intraoperative documentation did not allow for correlation between dose of neostigmine and postoperative clinical outcomes such as reintubation, pneumonia, or atelectasis. In addition, because the standard process of care at our centre, and many others, does not include monitoring of objective acceleromyography, the analysis cannot establish whether the dose of neostigmine is associated with complete reversal. Another limitation of our analysis is the inability to evaluate the impact of common medical comorbidities, the use of volatile anesthesia, or site of monitoring on the patterns of reversal.

Our study provides important information on current clinical patterns of use of neostigmine and exposes discrepancies between existing guidelines and actual clinical practice. In may be useful to incorporate our findings of potential NMB depth/reversal mismatch into design of future trials, as well as to influence future clinical guidelines, since patients in different BMI categories may require different interventions to improve safety of neuromuscular blockade.

## Conclusions

Contrary to our initial hypothesis, deeper levels of neuromuscular blockade were not associated with larger reversal doses. The median neostigmine dosing remained clinically similar across different levels of NMB as defined by the TOF counts. The median times to extubation following neostigmine administration were also similar for all TOF count categories. On the other hand, there was significant variability in neuromuscular blocker and neostigmine dosing across different BMI categories. The total median dose of neuromuscular blocking agents increased with increasing BMI, while the median dose of neostigmine decreased with increasing BMI.

## Abbreviations

AIMS: Anaesthesia Information Management System
ASA: American Society of Anaesthesiologists
BMI: Body mass index
ED95: Effect dose for which 95 % of the population exhibits the effect
EHR: Electronic health record
IBW: Ideal body weight
ICU: Intensive care unit
NMB: Neuromuscular blockade
OR: Operating room
SAS: Statistical Analysis System
SPSS: Software Package for Statistical Analysis
TBW: Total body weight
TOF: Train of four monitoring
UMHS: University of Michigan Health System
WHO: World Health Organization
